# Simultaneous Bilateral Tibial Tubercle Avulsion Fractures Complicated by Compartment Syndrome in an Adolescent With Osteogenesis Imperfecta: A Case Report

**DOI:** 10.1155/cro/1337169

**Published:** 2026-08-25

**Authors:** Tuckerman Jones, David Ahn, Rohan Suresh, Hyewon Kim, Sagie Haziza, Robert DalCortivo, Alexis Driscoll, Michael Metrione, Neil Kaushal

**Affiliations:** ^1^ Department of Orthopaedics, Rutgers New Jersey Medical School, Newark, New Jersey, USA, rutgers.edu

**Keywords:** case report, compartment syndrome, osteogenesis imperfecta, tibial tubercle, tibial tubercle avulsion fracture

## Abstract

**Background:**

Osteogenesis imperfecta (OI) is a rare collagen disorder causing brittle bones. Tibial tubercle fractures are uncommon in children, comprising < 2.7% of pediatric fractures, and bilateral injuries in OI are exceptionally rare. No prior reports detail bilateral tibial tubercle fractures with concurrent bilateral compartment syndrome. We present the first known case of this presentation in an 11‐year‐old boy.

**Case Presentation:**

An 11‐year‐old male with OI fell on his knees, presenting with severe bilateral leg pain. Imaging revealed bilateral Ogden III tibial tubercle fractures. Due to tense compartments, emergent fasciotomies were performed, followed by open reduction and internal fixation. At 16‐month follow‐up, the patient reported no complications and demonstrated normal ambulation.

**Conclusion:**

Surgeons should maintain a high index of suspicion for compartment syndrome in patients with OI presenting with tibial tubercle fractures, including after low‐energy mechanisms, and intervene promptly when clinically indicated.

## 1. Introduction

Osteogenesis imperfecta (OI) is a genetic disorder of collagen synthesis that results in increased bone fragility and susceptibility to fractures [1]. The incidence is approximately 1 in 20,000 [2]. Affected individuals are predisposed to frequent fractures despite minimal trauma [3]. Therefore, uncommon fractures are more prevalent in this population and often present in an uncharacteristic manner. Tibial tubercle fractures comprise between 0.4% and 2.7% of pediatric fractures and < 1% of all physeal injuries [4]. Although fractures are common in this population, tibial tubercle avulsion fractures remain rare, particularly in bilateral presentations. Due to the proximity of the recurrent anterior tibial artery, tibial tubercle fractures result in compartment syndrome have varied reported rates of incidence with studies showing up to 20% of cases [4, 5]. Currently, there are less than 10 reported cases of bilateral tibial tubercle fractures in patients with OI [4, 6–10].

Despite prior reports of bilateral tibial tubercle fractures and isolated cases of compartment syndrome, there is limited literature describing the combined presentation of bilateral tibial tubercle fractures with simultaneous bilateral compartment syndrome in patients with OI. This distinction is clinically important given the risk of delayed diagnosis, especially in cases with initially negative radiographs, and the potential for significant morbidity. Given the increased skeletal fragility associated with OI, these fractures may occur even after low‐energy mechanisms and may predispose patients to serious complications, including compartment syndrome. We present a rare case of an adolescent with OI who sustained bilateral tibial tubercle avulsion fractures complicated by bilateral compartment syndrome following a low‐energy mechanism, highlighting diagnostic challenges, management considerations, and implications for clinical practice.

## 2. Case Report

An 11‐year‐old male with a past medical history of OI Type 1 presented to our institution′s emergency department with bilateral lower extremity pain after directly falling onto both of his knees in gym class. Of note, he recently immigrated from Ecuador and has a history of 10 prior fractures that were managed nonoperatively. The patient had been receiving bisphosphonate therapy with zoledronic acid every 6–12 months in Ecuador through the Osteogenesis Imperfecta Foundation, beginning at approximately 2.5 years of age. However, therapy was discontinued approximately 2 years prior to presentation due to medication supply interruptions during the COVID‐19 pandemic. Otherwise, the patient is a healthy child that has met all developmental milestones and has had normal growth. The time from injury to presentation in the emergency department was approximately 2 hours. On arrival, vital signs were as follows: blood pressure, 102/57; heart rate, 86; respirations, 18; temperature, 36.9°C. The patient presented to the emergency department at approximately 2:30 p.m. following injury, initially without definitive signs of compartment syndrome. Over several hours, he developed progressively worsening pain and increasing compartment firmness, with clinical findings concerning for compartment syndrome becoming evident at approximately 9:00 p.m. despite escalation of pain control measures. Orthopedic surgery was subsequently consulted, and the patient was evaluated urgently with prompt operative intervention. On examination, the patient reported 10/10 pain in his bilateral lower extremities. He denied paresthesia and dysesthesia. Physical exam revealed bilateral lower extremity swelling and ecchymoses over the anterior proximal tibiae bilaterally. His anterior compartments were tense upon palpation. He was unable to straight leg raise and had limited active range of motion of his toes. He reported sensation to be intact to light touch globally. The patient endorses pain with passive stretch of the anterior compartment musculature bilaterally. The patient had palpable dorsalis pedal pulses and posterior tibial pulses. A detailed neurologic exam was limited due to pain.

**Figure 1:**
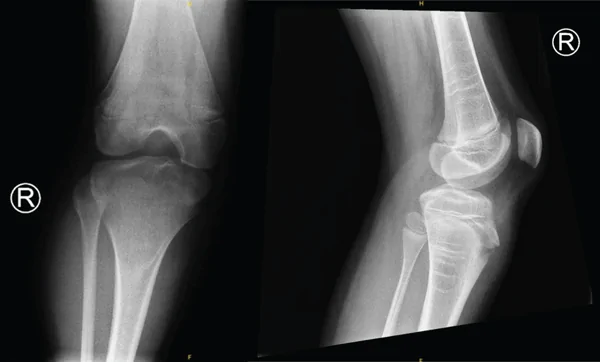
Right knee postinjury AP and lateral x‐rays.

**Figure 2:**
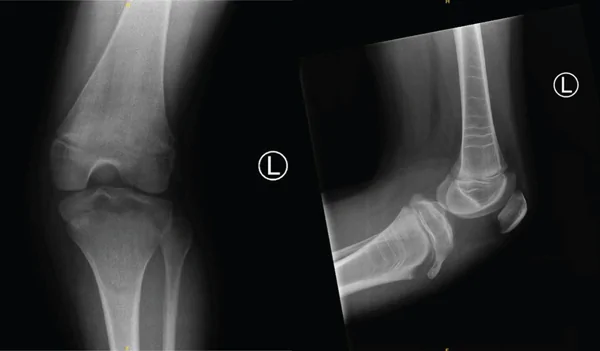
Left knee postinjury AP and lateral x‐rays.

**Figure 3:**
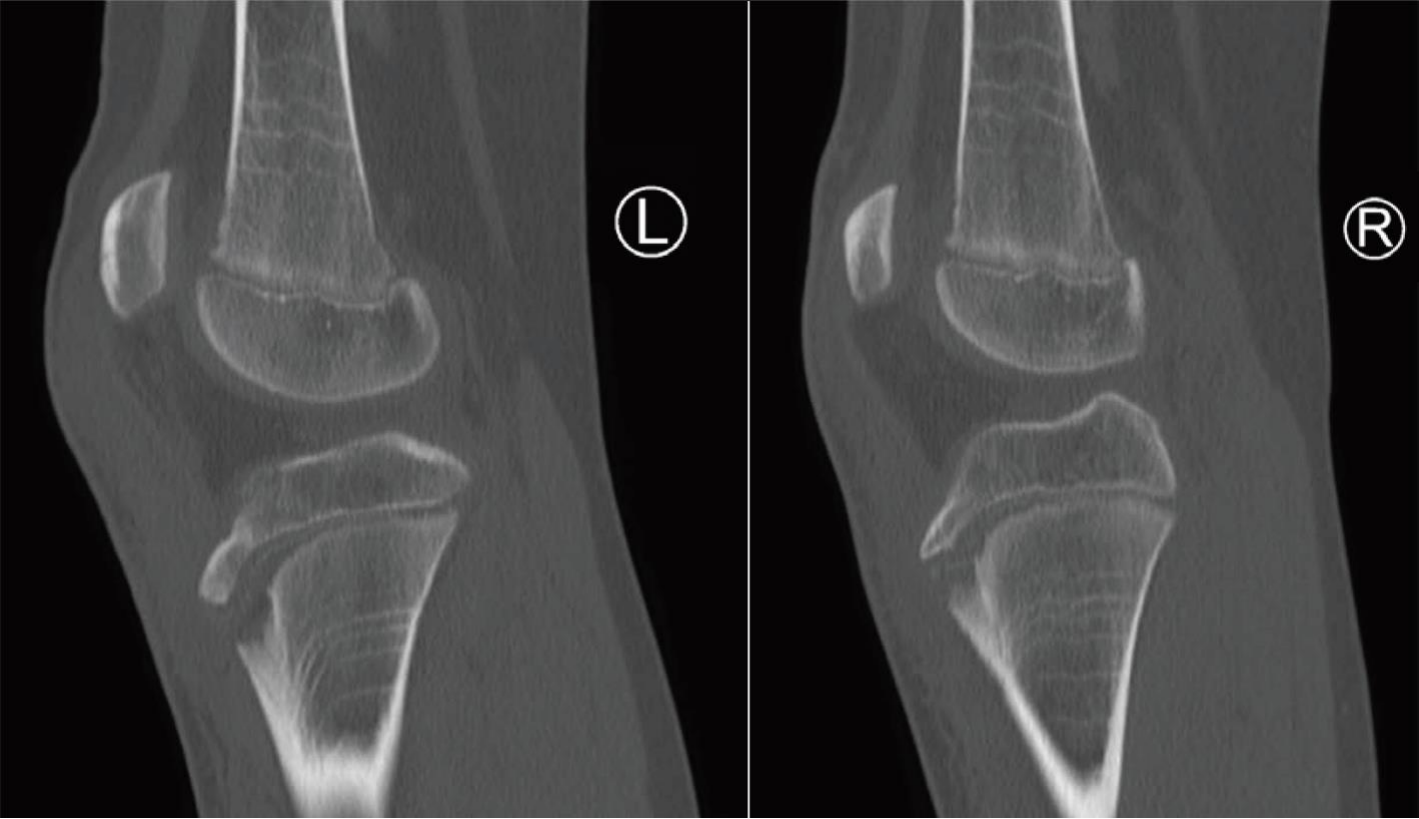
Bilateral knee CT scans.

Initial x‐rays of his bilateral tibia‐fibula were obtained and interpreted as negative for acute pathology or fractures (Figures 1 and 2). Despite the negative radiographs, the patient′s history of OI, persistent pain, inability to perform a straight‐leg raise, and concerning physical examination findings remained discordant with the imaging results. Given the continued clinical suspicion for significant injury, advanced imaging was obtained rather than relying solely on the initial radiographs. CT imaging was obtained and demonstrated bilateral tibial tubercle fractures extending into the proximal tibial physes (Figure 3).

**Figure 4:**
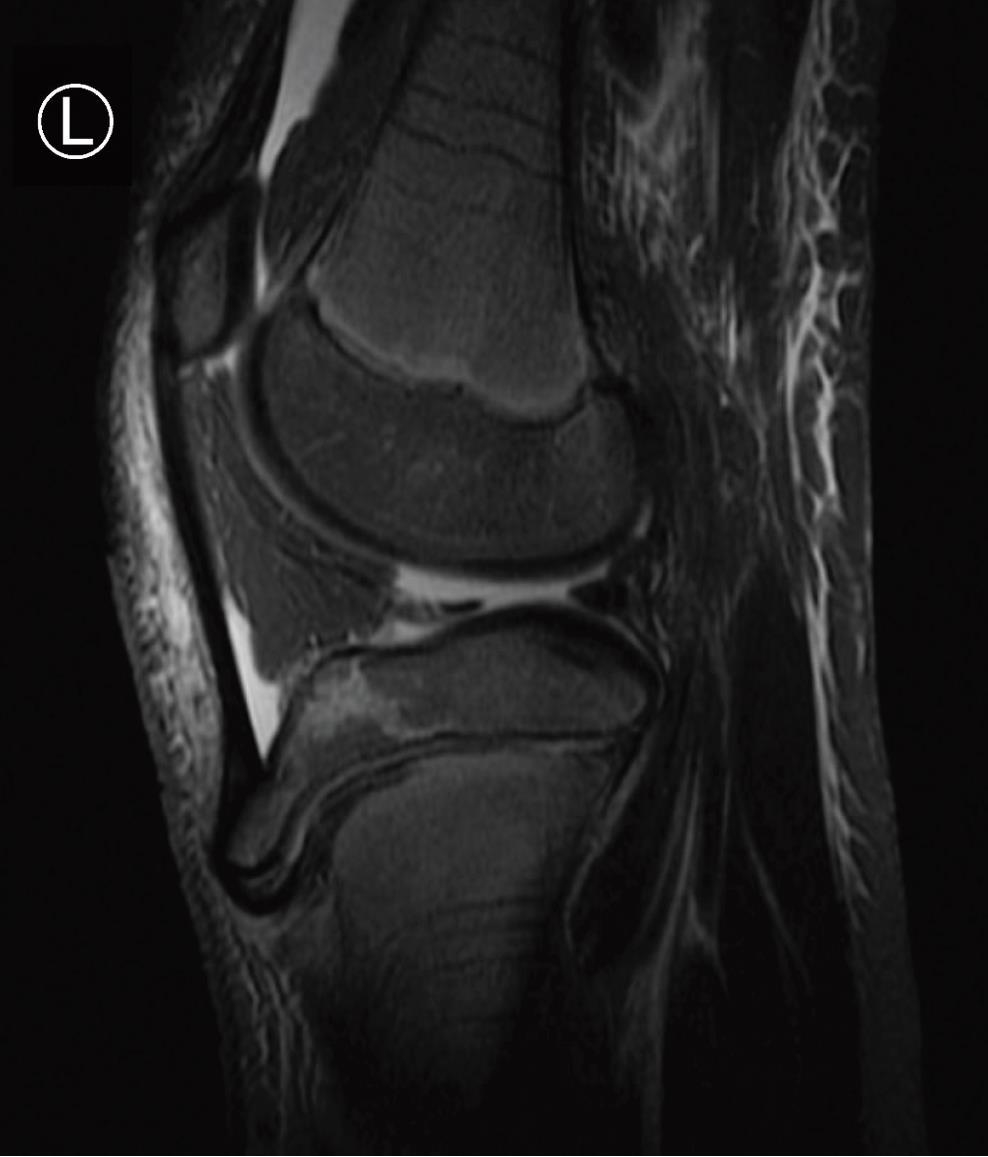
Left knee sagittal T2 MRI.

**Figure 5:**
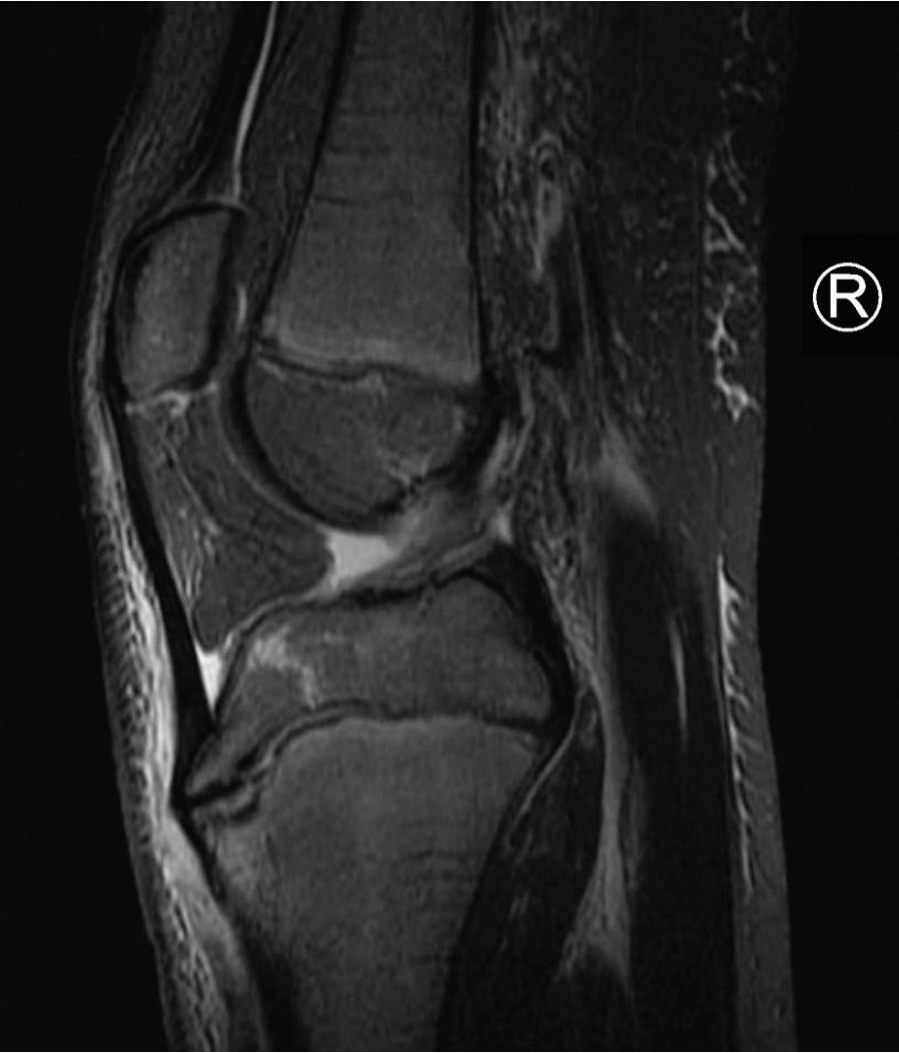
Right knee sagittal T2 MRI.

Given the patient′s tense anterior compartments and excruciating pain, the patient was sedated and compartment pressures were measured with Stryker monitor in the emergency department (Table 1). Despite compartment pressures meeting criteria only in the left lower extremity, the presence of severe bilateral pain, pain with passive stretch, tense compartments bilaterally, and inability to perform a detailed neurological examination prompted emergent bilateral anterior compartment fasciotomies. Intraoperative findings revealed significant damage to the anterior compartment musculature bilaterally, but muscle contractions were elicited with electrocautery stimulation. The lateral compartment musculature was viable bilaterally. Negative pressure wound dressings were applied bilaterally. Following emergent fasciotomy, MRI of the bilateral knees was obtained to better define fracture and soft tissue involvement. Given the planned return to the operating room for fasciotomy closure, fixation was performed in a staged manner, allowing for improved surgical planning and avoidance of an additional operative intervention. The left knee MRI was read as Ogden III tibial tubercle fracture. (Figure 4). The right knee MRI was read as an Ogden III tibial tubercle fracture (Figure 5). The patient was taken to the OR the following day for operative fixation of bilateral tibial tubercle fractures.

**Table 1:** Compartment pressures.

Compartment	Anterior	Lateral	Superficial posterior	Deep posterior
Right leg	31 mmHg delta: 50 mmHg	25 mmHg delta: 56 mmHg	20 mmHg delta: 61 mmHg	12 mmHg delta: 69 mmHg
Left leg	75 mmHg delta: 6 mmHg	28 mmHg delta: 53 mmHg	21 mmHg delta: 60 mmHg	18 mmHg delta: 63 mmHg
Diastolic pressure: 81 mmHg				

**Figure 6:**
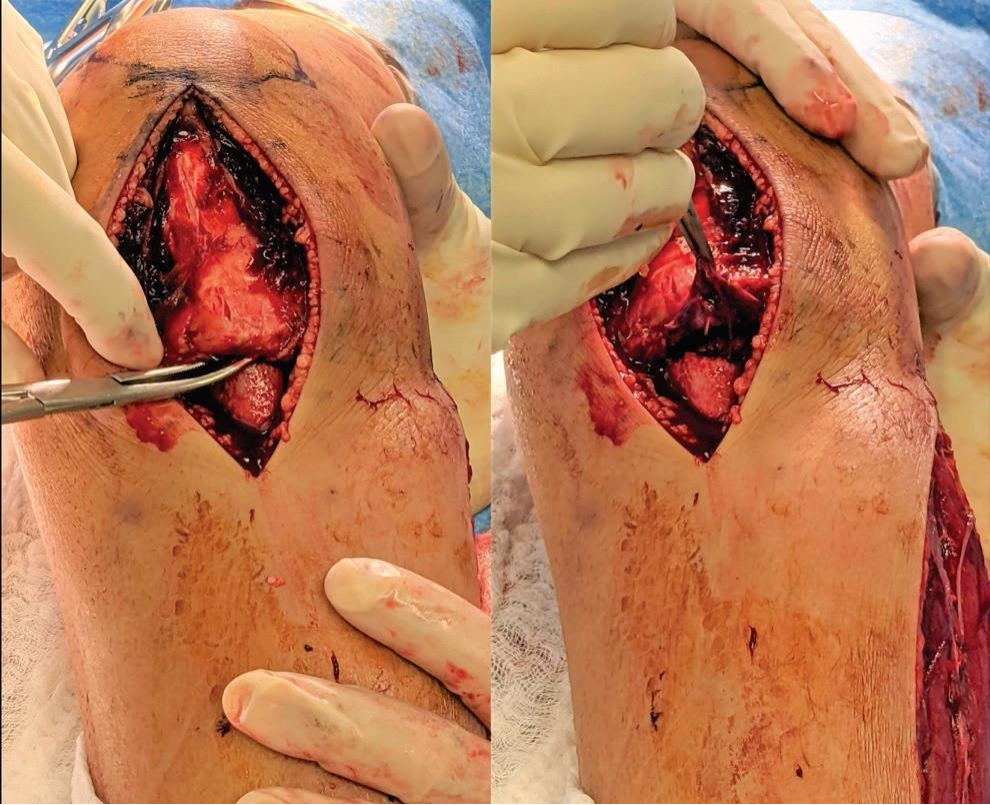
Intraoperative picture of left knee demonstrating patellar tendon entrapment under the tibial tubercle.

**Figure 7:**
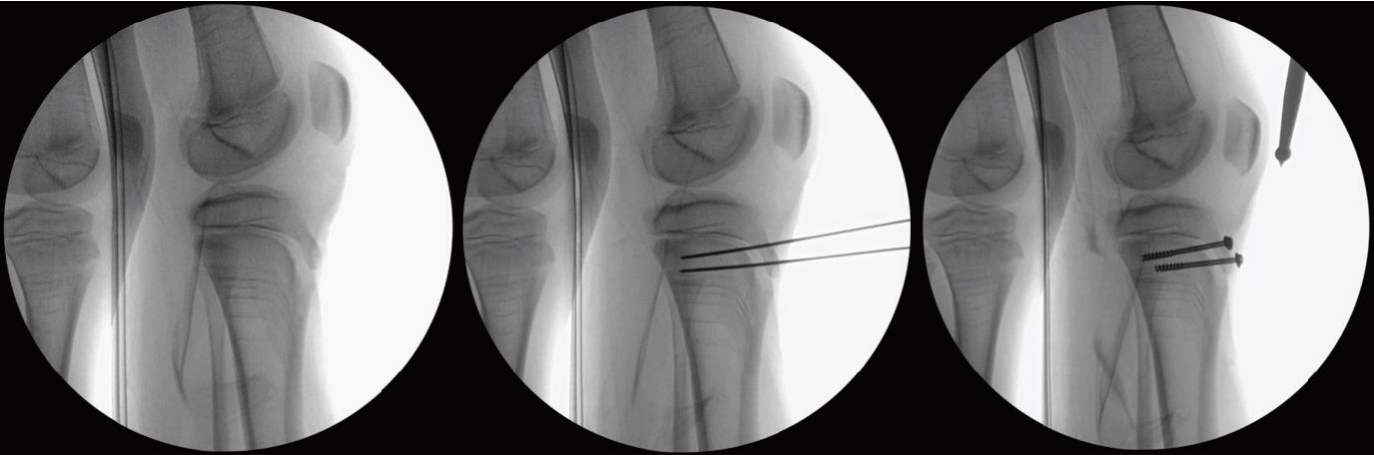
Left knee fluoroscopy images.

For the left knee, periosteal interposition within the fracture site was identified and cleared to allow reduction. The patellar tendon was observed intraoperatively to be displaced beneath the tibial tubercle fragment, a relationship not fully appreciated on preoperative imaging (Figure 6). The left tibial tubercle was found to be unstable. After obtaining reduction, two 4.0 mm partially threaded cannulated screws were placed to maintain fixation (Figure 7). For the right knee, the patellar tendon was found to be avulsed distally. Because the fracture was minimally displaced and stable, operative fixation of the patellar tendon distal to the tibial tubercle was achieved with a knotless suture anchor in order to restore the disrupted extensor mechanism.

**Figure 8:**
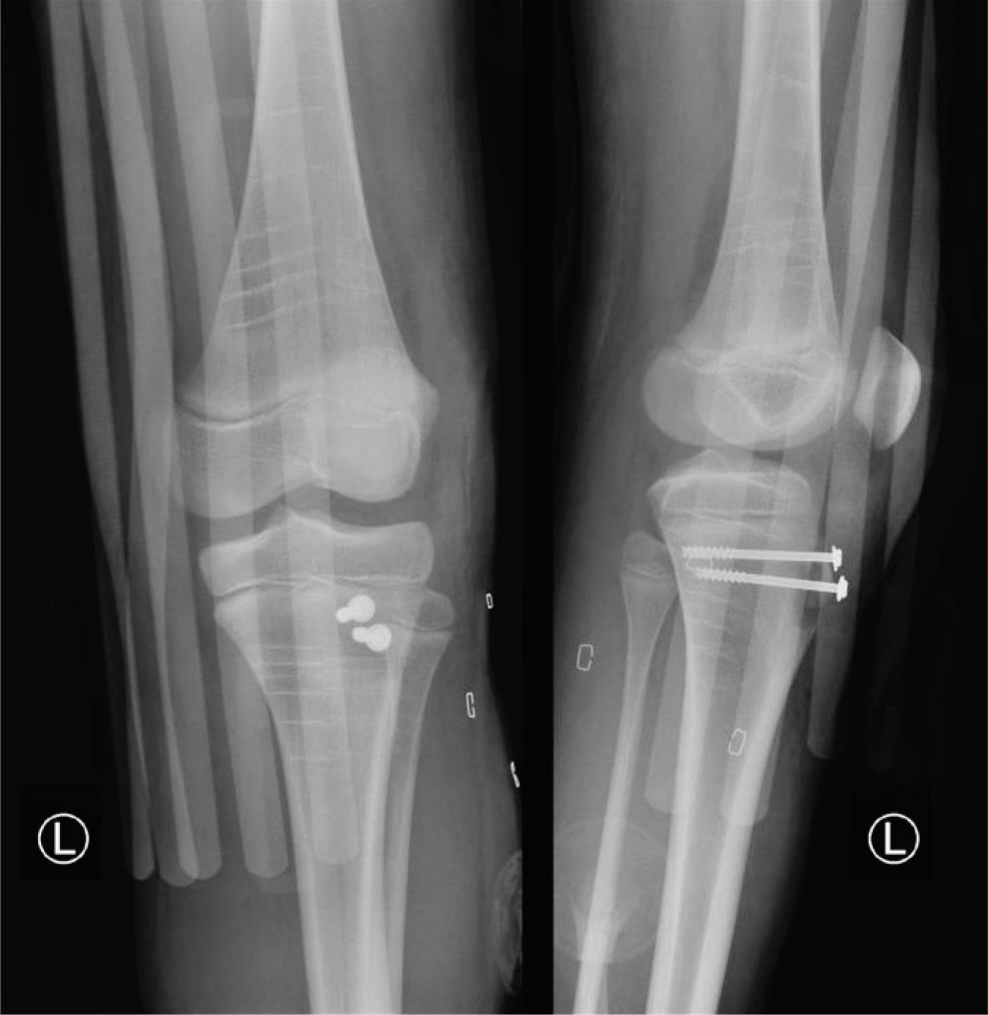
Left knee postoperative x‐rays.

**Figure 9:**
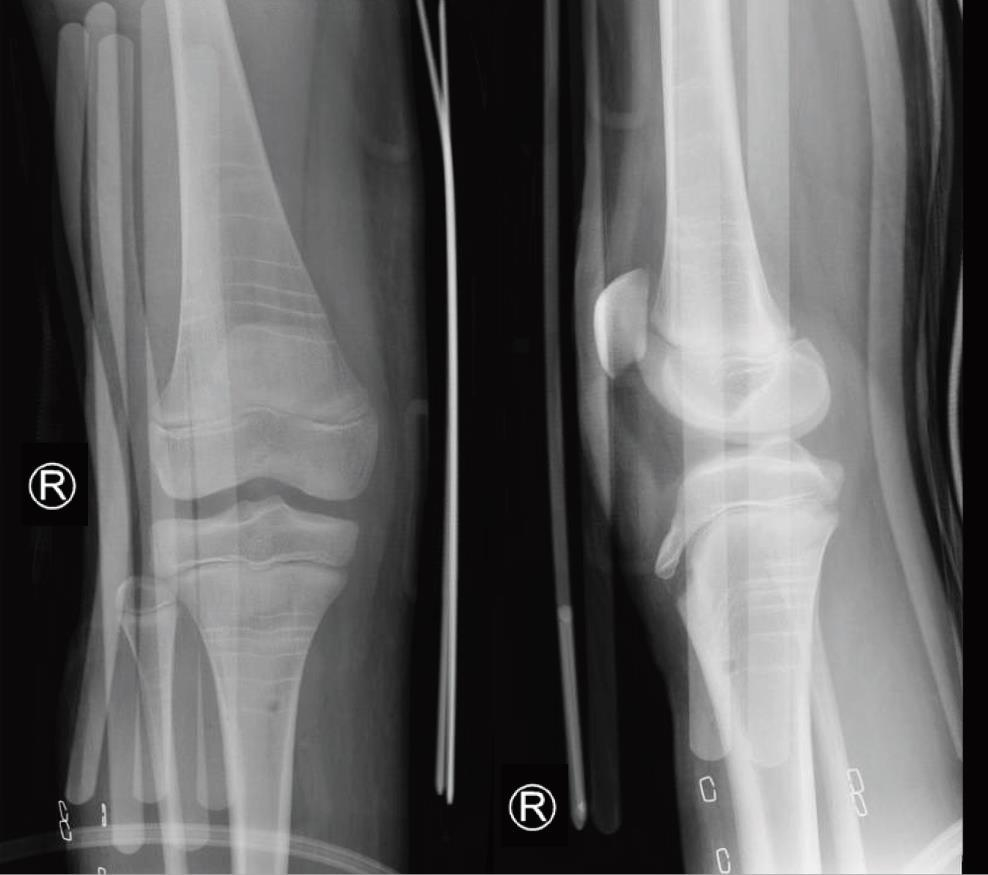
Right knee postoperative x‐rays.

Subsequently, three days after ORIF of bilateral tibial tubercle, the negative pressure wound dressing was removed, and the patient underwent primary closure of bilateral fasciotomy sites. Patient was placed in knee immobilizers, locked in extension (Figures 8 and 9), and made nonweight‐bearing to bilateral lower extremities. Postoperatively, the patient was made weight bearing as tolerated on the left lower extremity and nonweight‐bearing on the right and was provided with crutches for ambulation. At the 1‐month follow‐up, he was ambulating on both lower extremities. He subsequently underwent a structured rehabilitation program focused on progressive range of motion and extensor mechanism strengthening.

The patient′s most recent follow‐up was 16 months postprocedure. The patient reported no pain and no issues ambulating. Physical exam revealed soft compartments bilaterally. Fasciotomy and incision sites were clean, dry, and intact. Patient is able to straight leg raise bilaterally and has 5/5 strength of his bilateral extensor hallucis longus, flexor hallucis longus, tibialis anterior, and gastrocnemius‐soleus. Additionally, the patient had full ROM throughout his bilateral lower extremities and was noted to have a normal gait. He had intact sensation to light touch diffusely over his bilateral lower extremities. A structured timeline summarizing the patient′s injury, diagnostic evaluation, surgical management, and follow‐up course is presented in Table 2. This case report was prepared in accordance with the CARE (CAse REport) guidelines, and a completed CARE checklist is provided as supporting information.(Available here)

**Table 2:** Clinical timeline.

Time point	Clinical event
Injury	An 11‐year‐old male with osteogenesis imperfecta sustained a direct ground level fall onto both knees during gym class.
At 2 h postinjury	Presented to the emergency department without severe bilateral lower extremity pain.
Initial evaluation	Physical examination demonstrated bilateral swelling and ecchymosis about the bilateral knees and inability to ambulate.
Initial imaging	Bilateral tibia‐fibula radiographs were interpreted as negative for acute fracture.
Further imaging	Due to persistent clinical concern despite negative radiographs, CT imaging was obtained and demonstrated bilateral tibial tubercle fractures extending into the proximal tibial physes.
Over the next 8.5 h	Progressive worsening bilateral pain refractory to pain medication, pain with passive stretch and increasing compartment firmness raised concern for bilateral compartment syndrome. Orthopedics consulted.
At 8.5 h after initial presentation to ED	Orthopedics evaluates patient and bilateral compartment pressures were measured using a Stryker monitor.
Hospital Day 0	Emergent bilateral anterior compartment fasciotomies performed.
Hospital Day 1	MRI obtained demonstrating bilateral Ogden III tibial tubercle fractures.
Hospital Day 1	Open reduction and internal fixation of bilateral tibial tubercle injuries performed. Right‐sided patellar reconstruction performed.
Postoperative Day 3	Fasciotomy closure performed.
At 1‐month follow‐up	Ambulating on both lower extremities.
At 16‐month follow‐up	Pain‐free with full range of motion, normal gait, intact strength, and no complications.

## 3. Discussion

This case represents a rare presentation of bilateral tibial tubercle avulsion fractures complicated by bilateral compartment syndrome in a patient with OI. Although bilateral tibial tubercle fractures have been reported, and compartment syndrome is a known complication, the combination of these findings in a patient with OI is exceedingly uncommon.

There are fewer than 10 reported cases of bilateral tibial tubercle fractures in patients with OI. A list of documented cases is provided in Table 3. Khodadadyan‐Klostermann et al. [8] reported the first case in a 15‐year‐old male, followed by Tamborlane et al. [6], who described a second case in a 9‐year‐old male, marking the first instance with MRI documentation. Yotsuya et al. [7] later identified another case and found that the mean age of this injury in OI patients was 11.2 years. Andrews et al. [5] described a 14‐year‐old male with a unilateral tibial tubercle fracture complicated by compartment syndrome. Notably, all reported cases involve male pediatric patients between the ages of 9 and 15 [6–8], including the present case, suggesting a possible sex‐based predisposition that warrants further study. Remarkably, Yue et al. [11] reported the first known pediatric case of bilateral lower extremity anterior compartment syndrome following bilateral tibial tubercle fractures sustained through a high‐energy mechanism in a 15‐year‐old male. Tibial tubercle fractures can precipitate compartment syndrome due to hemorrhage, soft tissue swelling, and increased intracompartmental pressure, leading to neurovascular compromise. The initially negative radiographs in this case emphasize the risk of missed diagnosis, as tibial tubercle fractures may be subtle on initial imaging. Pandya et al. [12] demonstrated that up to 50% of these injuries are underestimated on lateral radiographs, with nearly 10% presenting with compartment syndrome or vascular compromise, reinforcing the need for high clinical suspicion and early advanced imaging when examination findings are concerning. The present case is notable for the occurrence of this injury pattern in a patient with OI following a low‐energy mechanism. This highlights that, in this population, even low‐energy trauma can result in this rare injury combination and lead to the potentially devastating complication of compartment syndrome. Despite elevated compartment pressures being elevated in only one of the affected limbs, concerning clinical signs of bilateral compartment syndrome such as severe bilateral pain, bilateral pain with passive stretch, tense compartments bilaterally, and inability to perform a detailed neurological examination prompted emergent bilateral anterior compartment fasciotomies. Although compartment pressure monitoring can provide valuable diagnostic information, this case underscores that clinical judgment remains paramount, particularly in emergent situations where delayed treatment may result in irreversible morbidity. This case demonstrates that given the fragility of bone and connective tissue in OI, these patients may be at even greater risk of this serious complication compared to the general population. Furthermore, collagen abnormalities in OI may contribute to increased vascular fragility and soft tissue susceptibility, which could theoretically predispose patients to greater risk of hemorrhage and elevated compartment pressures [13]. Compared with previously reported cases, this case underscores the importance of recognizing both osseous and soft tissue injury in patients with OI. The presence of periosteal interposition requiring surgical management further highlights the complexity of these injuries. Potential complications include growth disturbance, physeal arrest, and the possible need for hardware removal, all of which should be monitored during follow‐up. In pediatric patients with OI, tibial tubercle fractures carry a risk of compartment syndrome, and clinicians should maintain a high index of suspicion when concerning clinical symptoms arise, irrespective of the mechanism.

**Table 3:** Reported cases of tibial tubercle and proximal tibial injuries in pediatric patients.

Study	Year	Age (years)	Sex	Injury pattern	Mechanism	Laterality	Associated findings	Management	Outcome
Tamborlane et al.	2004	9	Male	Bilateral tibial tubercle avulsion fractures	Low energy	Bilateral	OI	Operative fixation	Good outcome
Khodadadyan‐Klostermann et al.	2003	15	Male	Bilateral tibial tubercle avulsion fractures	Low energy	Bilateral	OI	Operative fixation	Good outcome
Mehta et al.	2020	11	Male	Tibial tubercle avulsion + patellar tendon rupture	Low energy	Unilateral	OI, extensor mechanism disruption	Operative fixation and patellar tendon reconstruction	Good outcome
Yotsuya et al.	2023	10	Male	Repeated bilateral proximal tibial epiphyseal injuries	Minor trauma	Bilateral	OI	Operative management	Good outcome
Giunchi et al. Case #1	2012	14	Male	Bilateral tibial tubercle avulsion fractures	High energy	Bilateral	Extensor mechanism disruption	Operative fixation	Good outcome
Giunchi et al. Case #2	2019	14	Male	Bilateral tibial tubercle avulsion fractures + patellar tendon rupture	High energy	Bilateral	Extensor mechanism disruption	Operative fixation and bilateral patellar tendon reconstruction	Good outcome
Yue et al.	2019	15	Male	Bilateral tibial tubercle avulsion fractures	High energy	Bilateral	Compartment syndrome	Fasciotomy + immediate fixation	Good outcome
Present study	2026	11	Male	Bilateral tibial tubercle avulsion fractures + compartment syndrome	Low energy	Bilateral	OI, compartment syndrome	Fasciotomy + staged fixation	Good outcome

As a single case report, this study is limited in its ability to establish causality or guide treatment recommendations. However, given the rarity of bilateral tibial tubercle fractures with bilateral compartment syndrome in a patient with OI, the present case provides valuable clinical insight into the diagnosis and management of this injury pattern.

## 4. Conclusion

Bilateral tibial tubercle fractures with associated compartment syndrome are rare but carry significant morbidity. In pediatric patients with OI, this case demonstrates that such complications may occur even after low‐energy mechanisms. Recognizing the clinical signs of compartment syndrome in patients with OI following injury, regardless of mechanism, is critical, as early diagnosis and prompt intervention are essential to achieving favorable outcomes.

## Author Contributions

T.J., M.M., and N.K. conceived the study. D.A., R.S., H.K., S.H., A.D., and R.D. performed literature review and manuscript preparation. T.J., M.M., and N.K. supervised the project and provided critical revisions.

## Funding

No funding was received for this manuscript.

## Disclosure

All authors reviewed and approved the final manuscript.

## Ethics Statement

Institutional review board review determined that this single‐patient case report was exempt from formal IRB approval according to institutional policy.

## Consent

Patient consent was obtained from the patient′s parent/legal guardian.

## Conflicts of Interest

The authors declare no conflicts of interest.

## Supporting information


**Supporting Information** Additional supporting information can be found online in the Supporting Information section. File S1: Completed CARE checklist outlining adherence to the CARE reporting guidelines for case reports.

## Data Availability

The data that support the findings of this study are available from the corresponding author upon reasonable request.
